# Exploring the link between self-rated poor oral health and cardiovascular risk: a cross-sectional study using SCORE2

**DOI:** 10.1186/s12903-025-05671-6

**Published:** 2025-02-24

**Authors:** Peter Nymberg, Veronica Milos-Nymberg, Anton Grundberg, Nils Oscarson, Emelie Stenman, Kristina Sundquist

**Affiliations:** 1 Center for Primary Health Care Research, Department of Clinical Sciences Malmö, Lund University, Malmö, Sweden; 2 University Clinic Primary Care Skåne, Lund, Region Skåne Sweden; 3 Public Dental Service in Skåne, Lund, Sweden

**Keywords:** Self-rated oral health, Primary health care, Cross-sectional, Cardiovascular risk, Lifestyle habits

## Abstract

**Background:**

Poor oral health is associated with several non-communicable diseases including cardiovascular disease. There are also well-known associations between lifestyle habits, cardiovascular disease, and oral health. In Europe, SCORE2 is a recommended instrument for assessing an individual's risk of a cardiovascular event within 10 years. However, no previous studies have examined the association between self-rated oral health and SCORE2.

Using data from a cohort of 40- and 50-year-old individuals in Swedish primary healthcare, the present cross-sectional study investigated the association between self-reported poor oral health and cardiovascular risk assessed with targeted health dialogues and SCORE2.

**Methods:**

40- and 50-year-old individuals registered at 180 primary healthcare centres in southern Sweden were invited to participate in targeted health dialogues. Self-reported oral health and lifestyle habits were collected using a web questionnaire. Anthropometric measurements, blood pressure, and blood tests were collected. Data were analysed using group comparisons and regression models.

**Results:**

A total of 9499 individuals agreed to participate in the study and completed a targeted health dialogue between January 1, 2021, and January 10, 2024. The group who reported poor oral health had a higher proportion of high waist-hip ratio, insufficient physical activity, poor eating habits and tobacco use compared with individuals with good self-reported oral health. Significantly higher blood pressure was also noted, as well as elevated plasma glucose levels and low-density lipids. The regression analysis showed a significant association between poor self-rated oral health and cardiovascular risk by SCORE2 among both men and women. In the fully adjusted model, the association remained significant only for women.

**Conclusions:**

Our results indicate that individuals with poor self-rated oral health have higher cardiovascular risk and should be offered screening for unhealthy lifestyle habits to prevent cardiovascular events. We suggest that primary healthcare and dental care cooperate in identifying individuals at risk and intervene using evidence-based methods to prevent and address cardiovascular and oral diseases.

**Trial registration:**

ClinicalTrials.gov registration number. NCT04912739. Registration date 2021–06-03, retrospectively registered. Ethical approval was secured from the Swedish Ethical Review Authority (registration number 2020–02689 with subsequent amendments).

## Introduction

Poor oral health is a significant public health concern that affects millions worldwide. In addition, poor oral health is associated with several non-communicable diseases such as diabetes [1], cardiovascular disease [2], stroke [3], and Alzheimer's disease [4]. An association between oral health and cardiovascular disease (CVD) has been shown in different cohorts by using risk assessment models [5] and by assessing self-rated oral health (SROH) [6].

In Europe, the guidelines for cardiovascular prevention highlight the importance of using validated instruments in risk assessment [7]. At the beginning of the twenty-first century, the European Society of Cardiology developed the **S**ystematic COronar Risk Evaluation (SCORE) instrument to predict the 10-year risk of developing fatal cardiovascular disease among European individuals without prior CVD [8]. In 2021, SCORE2 was launched to update the original tool to estimate the 10-year risk for both fatal and non-fatal cardiovascular events [9]. However, neither SCORE nor SCORE2 have been used to examine the relation to oral health in well-defined cohorts.

### Factors affecting oral health

It is well-established that oral diseases, such as periodontitis and dental caries, are influenced by genetic factors and lifestyle habits [10]. A study of data from The National Health and Nutrition Examination Survey (NHANES) has indicated that healthier lifestyles, including non-smoking, moderate alcohol consumption, regular physical activity, and healthy sleep duration, tend to lower periodontitis incidence rates [11]. Conversely, those with unhealthy lifestyle patterns, including smoking [11, 12], excessive alcohol consumption [13], poor diet [14], low amount of physical activity, poor sleep patterns [15], and poor oral hygiene [16] are at higher risk of developing oral diseases. A cross-sectional study from Sweden has shown that lifestyle patterns differ not only by sex but also between native-born Swedes and foreign-born individuals [17], with a higher proportion of foreign-born individuals in Sweden reporting poor SROH [18].

### Oral health and the association with CVD risk factors

Studies have shown associations between oral diseases and the different variables included in SCORE2, such as elevated levels of low-density lipoprotein cholesterol (LDL) and reduced levels of high-density lipoprotein cholesterol (HDL), even after accounting for confounding variables such as diabetes and smoking among patients with periodontitis [19]. It has also been shown that patients with periodontitis have higher odds of developing hypertension with a linear relationship regarding the severity of periodontitis [18]. Another reason suggested as an independent risk factor for hypertension is poor oral hygiene, and the possible explanation is increased inflammation and impaired endothelial function [16]. Regarding smoking, there is a strong correlation between CVD as well as oral diseases, and studies have shown that smoking 15 cigarettes per day or more significantly increases the risk of developing oral diseases, i.e. periodontitis, among both men and women [16]. These findings underscore the high clinical relevance of oral health and CVD risk factors, such as lifestyle habits, and the relationship between these.

### Mapping cardiovascular risk factors

In Sweden, an evidence-based method has been developed to screen and improve the lifestyle habits connected with cardiovascular disease and diabetes in specific groups: Targeted Health Dialogues (THD). The systematic assessment determines the cardiovascular risk using a questionnaire about the individual's lifestyle behaviours, heredity for cardiovascular disease and diabetes combined with blood sampling, blood pressure measurement, waist-hip ratio, and body mass index. The results are visually presented in a colourful pedagogical tool such as a curve [20] or a star [21]. Using the visual pedagogical tool, a specially trained health dialogue coach discusses potential lifestyle improvements with the patient with the intention of reducing the risk of developing many diseases [22–25]. The habits included in THD affect the overall health and the risk of noncommunicable diseases such as hypertension [26], diabetes [27], adiposity [28], cardiovascular diseases [25, 29], and cancer [30]. THDs can identify individuals at risk for non-communicable diseases since they include lifestyle habits and objective health measures [29]. A recent review showed that according to GRADE (Grading of Recommendations, Assessment, Development and Evaluation), there was moderate evidence for the THD method regarding premature all-cause and cardiovascular mortality as well as for reduction in blood pressure, cholesterol, fasting blood sugar, waist measure, BMI, and improved dietary habits [31].

Using data derived from a cohort of 40- and 50-year-old individuals in Swedish primary healthcare, the present cross-sectional study investigated, for the first time, the association between self-reported poor oral health and the cardiovascular risk assessed with SCORE2.

## Method

### Participants and settings

Scania, which is situated in southern Sweden, encompasses both rural and urban areas and is home to 1.4 million residents. The population includes around 23% of foreign-born individuals from 179 countries [25]. Approximately 180 tax-funded private or public primary healthcare centres (PHCCs) provide the region's primary healthcare services. Since the beginning of 2021, due to a political decision, THDs have been implemented by the County Council at all PHCCs in Scania. As a result of this decision all 40- and 50-year-old individuals registered at these 180 PHCCs should be invited to a THD (50-year-olds were also invited from 2022 onwards). All THD participants were also invited to participate in the research project. This study is based on baseline data collected from recruited individuals between January 1, 2021, and January 10, 2024, who completed a THD and gave written consent to participate in the research study.

### Targeted Health Dialogue

Individuals aged 40- and 50 years were invited to a THD via a letter that included information about the screening process and an informed consent form for participating in a prospective cohort study. Before the THD, blood samples were collected through peripheral venipuncture and analysed for glucose, cholesterol, HDL, and LDL using routine clinical chemistry laboratory instruments.

Participants completed a detailed web-based questionnaire covering diet, physical activity, tobacco use, alcohol intake, oral health, mental stress, socioeconomic factors, psychosocial strain and family history of cardiovascular disease (CVD) or diabetes. We refer to previously published papers for a more detailed description of the THD method, including the questionnaire [17, 32, 33].

During the THD at the PHCC, BMI, waist-hip ratio, and blood pressure (mmHg) were measured.

### Measurements

SROH was assessed using a question with a five-graded scale: “How is your oral health?” The response alternatives were the following: very good, good, neither good nor poor, poor and very poor. In the analysis, the answers were dichotomised. Very good and good were considered as good; the other answers were considered as poor according to previous research on SROH [6, 18, 34].

The risk assessment criteria in the THDs for the various lifestyle habits were based on guidelines set by the Swedish National Board of Health and Welfare in 2018 and used in earlier studies describing THD [35].

The questionnaire contained two different physical activity questions. The first question was: How physically active are you in your leisure time? It was categorised into four response options: sedentary leisure time (mostly sitting), moderate exercise (equivalent to walking or cycling 4 h/week), strenuous exercise (equivalent to jogging or swimming 2 h/ week), and hard exercise (equivalent to running, swimming or competitive sports). Participants who chose sedentary leisure time were directly categorised as having insufficient physical activity. Participants who chose hard exercise were directly categorised as having a sufficient amount of physical activity. Participants who answered moderate or strenuous activity were asked to respond to follow-up questions about the mode of transport to work and other leisure time physical activities. Energy expenditure (kilocalories/week) is based on responses from the participants about time spent on leisure activities and commuting. The answers are then multiplied by an energy factor, and an average value of energy consumption per week is calculated. The threshold for insufficient physical activity was established at less than 2000 kilocalories per week in leisure activities [20, 36].

Dietary habits were assessed using questions about food quality, via a fat- and fibre index. The diet was categorised as 'Good' (healthy, no changes needed), 'Average' (some improvements needed), and 'Poor' (unhealthy, significant improvements needed). Participants reporting consumption of ‘sweets, chocolate, or sugar-sweetened drinks’ two or more times per day, or ‘cakes or cookies’ two or more times per day, or both categories once daily, were classified as having poor dietary habits, irrespective of the fat- and fibre index [37].

Alcohol intake was measured as standard glasses, with one standard glass equivalent to 12–15 cl of wine. High-risk alcohol consumption was defined as the intake of four or more standard glasses (for women) or six or more standard glasses (for men) of alcohol per week. Additionally, consuming four or more (for women) or five or more (for men) standard glasses per occasion at least once monthly was also deemed high-risk.

Tobacco use was divided into the following categories: no tobacco use, use of snus, e-cigarettes, hookah/water pipe, and use of cigarettes.

Socioeconomic factors (self-reported) relevant to the analysis included marital status (married or cohabiting, yes/no), educational level (≤ 9 years, 9–12 years (secondary school), > 12 years (post-secondary school)) and employment (employed/self-employed, unemployed, student, unpaid work or retired).

Sociodemographics regarding the place of birth were divided into three categories: Sweden, other European countries, and non-European countries.

The risk assessment in SCORE2 is based on an algorithm tailored to European populations based on the variables: age, sex, smoking status, systolic blood pressure, total cholesterol, HDL and four different geographic risk regions in Europe. Based on the assessment using SCORE2, an estimate of the individual 10-year risk of both fatal and non-fatal cardiovascular events can be calculated. SCORE2 applies to all individuals between 40- and 69 years and has a separate model for individuals older than 70 [9, 38].

SCORE2 risk categories were defined on three levels, i.e., Low risk: < 2.5% (40-year-olds), < 5% (50-year-olds); Medium risk: 2.5 to < 7.5% (40-year-olds), 5 to < 10% (50-year-olds); and High risk: ≥ 7.5% (40-year-olds), ≥ 10% (50-year-olds).

### Statistical analysis

The prevalence of cardiovascular risk factors among participants with good or poor self-rated oral health was described using means and standard deviations (SD) for continuous variables and numbers and percentages for categorical variables. Comparisons between groups were conducted using Pearson’s χ^2^ tests for categorical variables and Student’s t-test for continuous variables. The association between SROH, anthropometric measures and different lifestyle habits (independent variables) in the THDs and risk level of SCORE2 (dependent variable) was examined using ordinal logistic regression with proportional odds models [39]. We assessed the assumption of proportional odds using Brant tests, which were not significant. The results are shown as odds ratios (ORs), using a confidence interval (CI) of 95% and a significance level of 0.05. The association was stratified by sex and adjusted for educational level, place of birth and eating habits. We used a complete-case analysis, i.e., only those with data in the specific analysed variable were included. Confounders used in the regression analysis were selected based on previous knowledge about their association with oral health and CVD, birthplace, diet and using educational level as a proxy for socioeconomic status. All statistical analyses were performed with R version 4.4.0 (R Core Team, 2024).

## Results

**Fig. 1:**
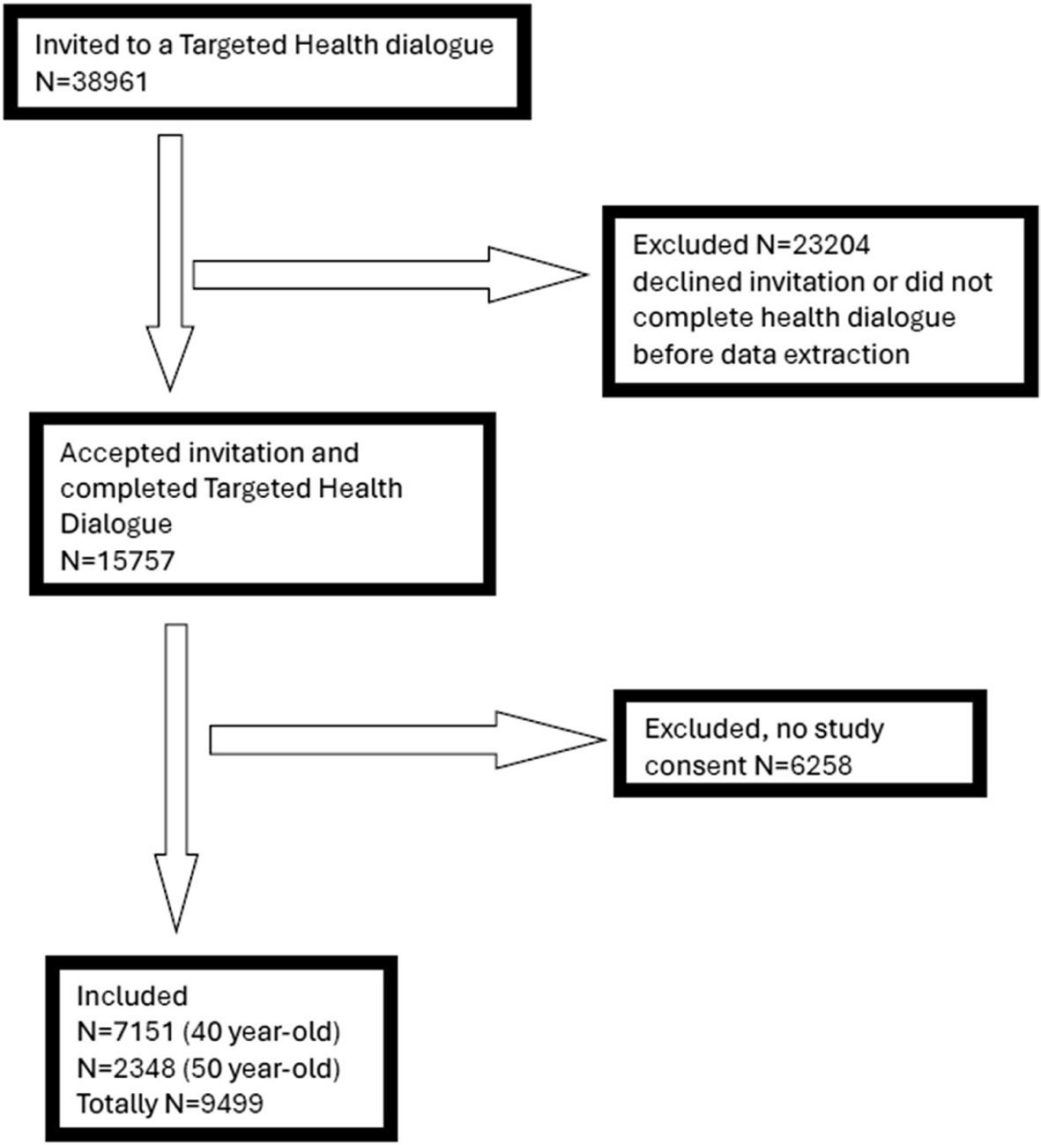
Flowchart over recruitment and dropout

A total of 38 961 individuals from 180 PHCCs were invited to a THD, of which 15 757 (40.4%) chose to participate. Of those, 9 499 (60.3%) completed a THD, provided written informed consent and were included in the analysis. Most of these (*n* = 7 451) were 40 years of age (Table 1). A flow chart of the inclusion is presented in Fig. 1.

**Table 1:** Sex, age and socioeconomic factors are self-reported. Data are presented as n (%). The percentage is presented per row

**Characteristic**	**Total, *n* = 9499**^b^	**Good oral health**	**Poor oral health**	***p*-value**^a^
Oral Health		6810 (71.9)	2666 (28.1)	-
Age				0.18
40 years	7151	5101 (71.5)	2032 (28.5)	
50 years	2348	1709 (72.9)	634 (27.1)	
Sex				< 0.001
Men	4240	2881 (68.1)	1351 (31.9)	
Women	5259	3929 (74.9)	1315 (25.1)	
Level of education				< 0.001
≤ 9 years	567	298 (52.7)	268 (47.3)	
Secondary school	2772	1828 (66.1)	938 (33.9)	
Post-secondary school	6133	4672 (76.2)	1457 (23.8)	
Missing^c^	27	12 (80.0)	3 (20.0)	
Place of birth				< 0.001
Sweden	6599	4988 (75.7)	1605 (24.3)	
Other European country	1196	803 (67.3)	391 (32.7)	
Non-European country	1678	1005 (60.0)	670 (40.0)	
Missing^c^	26	14 (100)	0 (0.0)	
Married/cohabitant				< 0.001
Yes	7646	5568 (72.9)	2067 (27.1)	
No	1829	1231 (67.4)	596 (32.6)	
Missing^c^	24	11 (78.6)	3 (21.4)	
Employment				< 0.001
Employed/Self-employed	8627	6321 (73.4)	2295 (26.6)	
Unemployed	463	255 (55.3)	206 (44.7)	
Student	225	138 (61.3)	87 (38.7)	
Unpaid work	54	33 (61.1)	21 (38.9)	
Retired	81	38 (46.9)	43 (53.1)	
Missing^c^	49	25 (64.1)	14 (35.9)	

^a^*P*-value comparing the characteristics of good and poor oral health was calculated using Pearson’s χ^2^ tests for categorical variables

^b^23 individuals (0.2%) have missing oral health data

^c^Missing refers to the specific category

### Health Metrics and Lifestyle Variations

There were no statistically significant differences regarding the distribution of poor or good SROH between 40- or 50-year-olds (p = 0.18). However, there were significant differences in the distribution of those reporting poor SROH regarding sex, educational level, country of origin, marital status and employment status (Table 1).

The group with poor SROH had a significantly higher mean value of both systolic (124.4 vs 123.3, p < 0.001) and diastolic (80.7 vs 79.6, p < 0.001) blood pressure compared with the group with good SROH. They also had lower mean HDL, higher LDL, higher fasting plasma glucose and BMI (Table 2).

**Table 2:** Anthropometric measures and lifestyle habits compared with Self-rated oral health. Data are presented as n (%). The percentage is per column

**Characteristic**	**Total**^c^	**Good oral health *n* = 6810**	**Poor oral health *n* = 2666**	***p*-value**^**a**^
Systolic blood pressure, (mmHg) mean (SD)	123.6 (14.9)	123.3 (14.8)	124.4 (15.1)	0.001
Diastolic blood pressure, (mmHg) mean (SD)	79.9 (10.5)	79.6 (10.4)	80.7 (10.7)	< 0.001
Blood pressure level, n (%)				< 0.001
Normal (< 130/85)	5387 (56.7)	3955 (58.1)	1420 (53.3)	
High normal (130–139/85–89)	1953 (20.6)	1371 (20.1)	580 (21.8)	
High (≥ 140/90)	2128 (22.4)	1470 (21.6)	654 (24.5)	
Missing^d^	31 (0.3)	14 (0.2)	12 (0.5)	
Total cholesterol, (mmol/L) mean (SD)	4.80 (0.89)	4.81 (0.89)	4.78 (0.90)	0.25
HDL cholesterol, (mmol/L) mean (SD)	1.44 (0.49)	1.46 (0.47)	1.38 (0.51)	< 0.001
LDL cholesterol, (mmol/L) mean (SD)	3.28 (0.92)	3.26 (0.92)	3.32 (0.92)	0.002
LDL cholesterol, n (%)				0.14
< 5 mmol/l	9027 (95.0)	6486 (95.2)	2523 (94.6)	
≥ 5 mmol/l	415 (4.4)	285 (4.2)	130 (4.9)	
Missing^d^	57 (0.6)	39 (0.6)	13 (0.5)	
F-plasma glucose, (mmol/L) mean (SD)	5.46 (0.82)	5.42 (0.75)	5.55 (0.98)	< 0.001
F-plasma glucose, n (%)				< 0.001
≤ 6 mmol/l	8464 (89.1)	6140 (90.2)	2307 (86.5)	
6.1 – 6.9 mmol/l	742 (7.8)	491 (7.2)	250 (9.4)	
≥ 7 mmol/l	206 (2.2)	119 (1.7)	87 (3.3)	
Missing^d^	87 (0.9)	60 (0.9)	22 (0.8)	
BMI, mean (SD)	26.6 (5.0)	26.3 (4.8)	27.5 (5.4)	< 0.001
BMI, n (%)				< 0.001
< 25	4077 (42.9)	3124 (45.9)	948 (35.6)	
25 – 29.9	3390 (35.7)	2382 (35.0)	1000 (37.5)	
≥ 30	1996 (21.0)	1280 (18.8)	711 (26.7)	
Missing^d^	36 (0.4)	24 (0.4)	7 (0.3)	
Waist-hip ratio, n (%)				< 0.001
Normal	5602 (59.0)	4290 (63.0)	1303 (48.9)	
High	3786 (39.9)	2450 (36.0)	1327 (49.8)	
Missing^d^	111 (1.2)	70 (1.0)	36 (1.4)	
SCORE2^b^, n (%)				< 0.001
Low risk	8314 (87.5)	6088 (89.4)	2208 (82.8)	
Medium risk	1058 (11.1)	645 (9.5)	413 (15.5)	
High risk	41 (0.4)	20 (0.3)	21 (0.8)	
Missing^d^	86 (0.9)	57 (0.8)	24 (0.9)	
Physical activity, n (%)				< 0.001
Sufficient	3076 (32.4)	2358 (34.6)	715 (26.8)	
Insufficient	6165 (64.9)	4294 (63.1)	1861 (69.8)	
Missing^d^	258 (2.7)	158 (2.3)	90 (3.4)	
Eating habits, n (%)				< 0.001
Good (healthy)	2954 (31.1)	2191 (32.2)	763 (28.6)	
Average	2527 (26.6)	1892 (27.8)	630 (23.6)	
Poor (unhealthy)	2497 (26.3)	1642 (24.1)	849 (31.8)	
Missing^d^	1521 (16.0)	1085 (15.9)	424 (15.9)	
Alcohol consumption, n (%)				0.31
Low-risk consumption	7401 (77.9)	5312 (78.0)	2079 (78.0)	
High-risk consumption	1610 (16.9)	1176 (17.3)	432 (16.2)	
Missing^d^	488 (5.1)	322 (4.7)	155 (5.8)	
Tobacco use, n (%)				< 0.001
None	7552 (79.5)	5724 (84.1)	1816 (68.1)	
Snus, e-cigarettes, hookah/waterpipe	1153 (12.1)	704 (10.3)	449 (16.8)	
Cigarettes	698 (7.3)	325 (4.8)	372 (14.0)	
Missing^d^	96 (1.0)	57 (0.8)	29 (1.1)	

^a^*P*-value comparing the characteristics of good and poor oral health was calculated using Pearson’s χ^2^ tests for categorical variables and Student’s t-test for variables represented by mean (SD), and Chi-squared test for variables represented with n (%)

^b^SCORE2 risk categories are defined as Low risk = < 2.5% (40-year-olds), < 5% (50-year-olds). Medium risk = 2.5 to < 7.5% (40-year-olds), 5 to < 10% (50-year-olds). High risk = ≥ 7.5% (40-year-olds), ≥ 10% (50-year-olds)

^c^23 individuals (0.2%) have missing oral health data

^d^Missing refers to the specific category

Among those with poor SROH, there was a higher proportion of individuals with a BMI over 25 (64.2%) compared to those with good SROH (53.8%) (p < 0.001). This group also had a significantly higher proportion of individuals with a high waist-hip ratio (WHR) (63.0%) compared with individuals with good SROH (36.0%) (Table 2).

Significant group differences in lifestyle habits were found, with a higher proportion reporting insufficient physical activity, poor eating habits, or using tobacco in the group with poor SROH (p < 0.001). There were no significant group differences regarding alcohol consumption (p = 0.31) (Table 2).

There was a higher proportion of participants with medium or high CVD risk measured with SCORE2 among those who reported poor SROH compared with individuals considering SROH as good. (p < 0.001) (Table 2).

### Association between SROH and Cardiovascular Risk

A logistic regression analysis was performed to study the association between poor SROH and an increase in the SCORE2 risk level. In the regression models, the analysis showed a high odds ratio (OR) for increased cardiovascular risk according to SCORE2 if SROH was poor among men, OR 1.42 (95% CI 1.23–1.65) and for women, OR 4.50 (95% CI 2.93–7.01) in the crude model. When adjusted for education and place of birth, the OR decreased both among men (OR 1.23, 95% CI 1.06–1.43) and women (OR 3.93, 95% CI 2.52–6.20) but still showed a significantly elevated risk assessment according to SCORE2 if SROH was poor. In the fully adjusted model, where eating habits were added, the significant OR disappeared among men (OR 1.17. 95% CI 0.99–1.38) but not among women, where the odds ratio increased compared with the crude and the second adjusted model (OR 4.96 95% CI 2.91–8.69) (Table 3).

**Table 3:** The odds ratio of increased risk level in SCORE2 for participants with self-rated oral health rated as poor

Poor oral health	All, OR (95% CI)	*p*-value	Men, OR (95% CI)	*p*-value	Women, OR (95% CI)	*p*-value
Model 1^a^	1.80 (1.58–2.05)	< 0.001	1.42 (1.23–1.65)	< 0.001	4.50 (2.93–7.01)	< 0.001
Model 2^b^	1.52 (1.33–1.74)	< 0.001	1.23 (1.06–1.43)	0.008	3.93 (2.52–6.20)	< 0.001
Model 3^c^	1.45 (1.25–1.68)	< 0.001	1.17 (0.99–1.38)	0.068	4.96 (2.91–8.69)	< 0.001

Assessed using ordinal logistic regression with categorical SCORE2 as a dependent variable

^a^Model 1 = unadjusted

^b^Model 2 = adjusted for education and place of birth

^c^Model 3 = Model 2 + adjusted for eating habits

## Discussion

Our results show that the group with poor SROH had a significantly higher proportion of self-reported unhealthy lifestyle habits and cardiovascular risk factors, and a higher socioeconomic strain compared with those reporting good SROH. The result of the regression analysis showed a positive association between poor SROH and an increased risk level according to SCORE2 in both men and women. After adjustments for education and place of birth, there was a reduction of the ORs in both men and women, indicating that these socio-demographic characteristics may influence the cardiovascular risk according to SCORE2 in individuals with poor SROH. The significant association disappeared among men when we adjusted for dietary intake. Among the participants with missing diet data, there were more women than men. This might explain why the association between SROH and SCORE levels disappeared for men but became stronger for women. Our findings are in line with previous research studying the association between periodontitis and the risk of cardiovascular events [5]. The analysis also showed significant associations between poor SROH and socioeconomic factors, unhealthy lifestyle habits, and blood lipids, matching results from previous research [11–17]. Opposite to the present study, many previous studies were based on oral examinations where periodontitis was verified and often classified [5, 40, 41]. Since SROH was linked to unhealthy lifestyle habits, poor oral health may be a possible marker concerning the risk of unhealthy lifestyle habits. In the future, collaboration between dentists and primary care may be a way to identify vulnerable individuals and refer them for health interventions, potentially reducing their future cardiovascular risk. This study was not able to reveal any causal relationship between SROH and SCORE2, but our findings expose the shared risk factors that link them (Table 2). Patients should be recommended dental care if poor dental health is identified during lifestyle and risk factor screening in primary care, and dentists should recommend such screening if poor oral health is observed. Improved oral health can enhance an individual's dietary intake as healthier foods, which are sometimes more challenging to chew, become more accessible. This may lead to a decreased risk of incident CVD and further improvements in oral health [42]. High LDL levels are associated with an increased risk of cardiovascular disease as well as periodontitis; however, even if the periodontitis is treated, the increased level of LDL remains [16]. The reason may depend on the fact that the dietary intake has not changed after the treatment of periodontitis. This possible explanation can also be appropriate to smoking, which is one of the leading causes of CVD and affects oral health. To lower the risks of both CVD and poor SROH, smoking cessation is required. Therefore, we suggest that primary healthcare and dental care should cooperate in identifying individuals at risk and intervene using evidence-based methods such as THDs to prevent cardiovascular and oral diseases.

In Sweden, healthcare is publicly funded through general taxation, resulting in low patient costs. Dental care costs may decrease the willingness to attend a dentist appointment, even with a referral. After the age of 24, dental care becomes more expensive, and the fixed annual allowance for dental care in Sweden is often insufficient [43].

Upcoming studies may benefit from focusing specifically on oral health, lifestyle habits and SCORE2 in vulnerable groups (such as individuals with mental illness [32, 41, 42]), which is in line with a report from the Swedish Agency for Health Technology Assessment and Assessment of Social Services (in Swedish, SBU) [44].

### Strengths

To the authors’ knowledge, this is the first study that investigates the association between SROH and cardiovascular risk factors assessed with THDs and SCORE2 in a well-defined population of 40- and 50-year-old men and women. The study is based on a population with high demographic diversity (including socioeconomic characteristics) and covers a large geographic area (rural, suburban and urban), thus increasing the generalisability of the results. The recruitment is based on an implemented method in primary care (THD) offered to all individuals in specific age groups. A major strength of this study is the assessment of cardiovascular risk using objective measurements such as blood samples, blood pressure, and anthropometric data. Another strength is the low amount of missing information in the collected material, with only 0.2% not answering the question about SROH and with similar proportions of missing data for the variables included in the calculation of SCORE2.

### Limitations

The study's cross-sectional nature does not allow for establishing a causal association between SROH and cardiovascular risk. Another limitation is that SROH was self-reported and not objectively measured, which implies a risk of self-reporting bias. This bias may have affected the results and the high OR in the analysis. However, SROH has previously been shown to be strongly associated with questionnaires on oral hygiene behaviours and pathological findings in oral examinations [45]. One limitation is that the number of participants with missing data in the diet category was relatively high, which may have slightly affected the adjustment for diet in the logistic regression. In addition, the potential risk of selection bias should be acknowledged. Since only a quarter of the invited individuals agreed to participate, those who accepted might have been more interested in their cardiovascular and overall health and, thus, more likely to change their unhealthy habits. Therefore, there is a risk of selection bias (healthy cohort bias). Meanwhile, more vulnerable groups, such as socially deprived individuals and those with mental illness or substance abuse disorders, are more likely to experience poor oral health and are often underrepresented in health surveys [46]. If the cohort had only consisted of participants from different vulnerable groups, the results might have shown other associations between SROH, SCORE2, and other measured parameters included in the analysis.

Another major limitation decreasing the generalisability is the inclusion of specific age groups (40- and 50-year-olds), which limits the application of the results to other age groups.

## Conclusions

Our results indicate that individuals with poor SROH also have higher cardiovascular risk and should preferably be offered a screening for cardiovascular risk factors and unhealthy lifestyle habits when dental problems are identified. We suggest that primary healthcare and dental care should cooperate in identifying individuals at risk and intervene using evidence-based methods, such as THDs, to prevent and address cardiovascular and oral diseases.

## Data Availability

Data will be made available upon reasonable request.

## Abbreviations

CVD: Cardiovascular disease
CI: Confidence interval
GRADE: Grading of Recommendations, Assessment, Development and Evaluation
HDL: High-density lipoprotein cholesterol
LDL: Low-density lipoprotein cholesterol
NHANES: National Health and Nutrition Examination Survey
OR: Odds ratio
PD: Periodontal disease
PHCCs: Primary Health Care Centres
SCORE: Systematic COronary Risk Evaluation
SROH: Self-rated oral health
SD: Standard deviation
THD: Targeted Health Dialogues
TG: Triglycerides
WHR: Waist-hip ratio
